# Photic Maculopathy Caused by a Laser Pointer

**DOI:** 10.7759/cureus.94249

**Published:** 2025-10-09

**Authors:** Gagandeep Naral, Shikha B Shetty, Sripathi Kamath, Madhurima Nayak

**Affiliations:** 1 Ophthalmology, Father Muller Medical College, Mangalore, IND

**Keywords:** 650 nm, central scotoma, laser, optical coherence tomography, photic maculopathy

## Abstract

Laser pointers are easily available devices and are frequently used for teaching and presentation purposes. We present a case of photic maculopathy in a man in his 20s who came with central scotoma and blurred vision in the right eye following accidental exposure to a red laser pointer. Visual acuity of the affected eye was 20/30 and N8. On examination of the fundus, a yellowish foveal lesion was seen. The Amsler grid test revealed a central scotoma. Fundus autofluorescence showed hyperfluorescence with central hypofluorescence. A diagnosis of photic maculopathy was made based on characteristic findings on optical coherence tomography. On follow-up, the size of the lesion decreased. However, the patient complained of a persisting scotoma. This case highlights the public health importance of creating awareness among people regarding the strict regulations on the unscrupulous use of laser pointers to prevent vision loss.

## Introduction

Laser stands for light amplification by stimulated emission of radiation, and as the name suggests, it functions by emitting a stimulated beam of radiation for light amplification. Laser pointers have been utilized in day-to-day life not only for business and educational presentations and medical applications but also for construction purposes, astronomy, military use, scientific research, and industrial applications. The common wavelengths of pointers available in the market vary from 532 nm to 670 nm. Lasers can cause photocoagulation, photo-disruptive, and photochemical damage. As the eye is designed to focus bright light onto the macula, it is the commonest site for laser-induced damage. Furthermore, the type of laser used may also influence the type of injury it can cause. Ocular injuries range from subtle retinal pigment epithelium (RPE) abnormalities, vitelliform-like lesions, full-thickness macular holes, and choroidal neovascular membranes [1]. Photic maculopathy is one of them. 

Photic maculopathy, also known as solar maculopathy and eclipse retinopathy, is a result of photochemical damage to the retinal tissues and is characterized by the focal disruption of outer retinal layers. However, there has been a rise in the incidence of retinal injuries caused by laser pointers [2]. Given the severity of accidental exposure to lasers, the public health issues that arise with the use of this equipment are significant. 

## Case presentation

A moderately built, apparently healthy man in his 20s presented with complaints of a sudden onset of seeing a black, blurry spot in his right eye after accidental exposure to a laser pointer by his neighbour. The exposure occurred during the daytime when the patient was engaged in conversing with his neighbour, who demonstrated the laser pointer and accidentally shone it into the patient's eye. He was at a distance of around 2 m, and the exposure lasted for no more than two seconds. He described it as a "red-colored light". He complained of difficulty in reading, recognizing faces, and performing tasks requiring precision following the exposure. The patient complained that the beam unintentionally entered his right eye while using a laser pointer. The pointer had a red laser of wavelength 650 nm and an output power of 5 mW. The patient did not complain of any associated pain, redness, or watering.  He did not have any history of decreased vision in either eye before this incident and had no history of use of spectacles. He had no complaints in the left eye or significant past medical or ocular history.  

**Figure 1:**
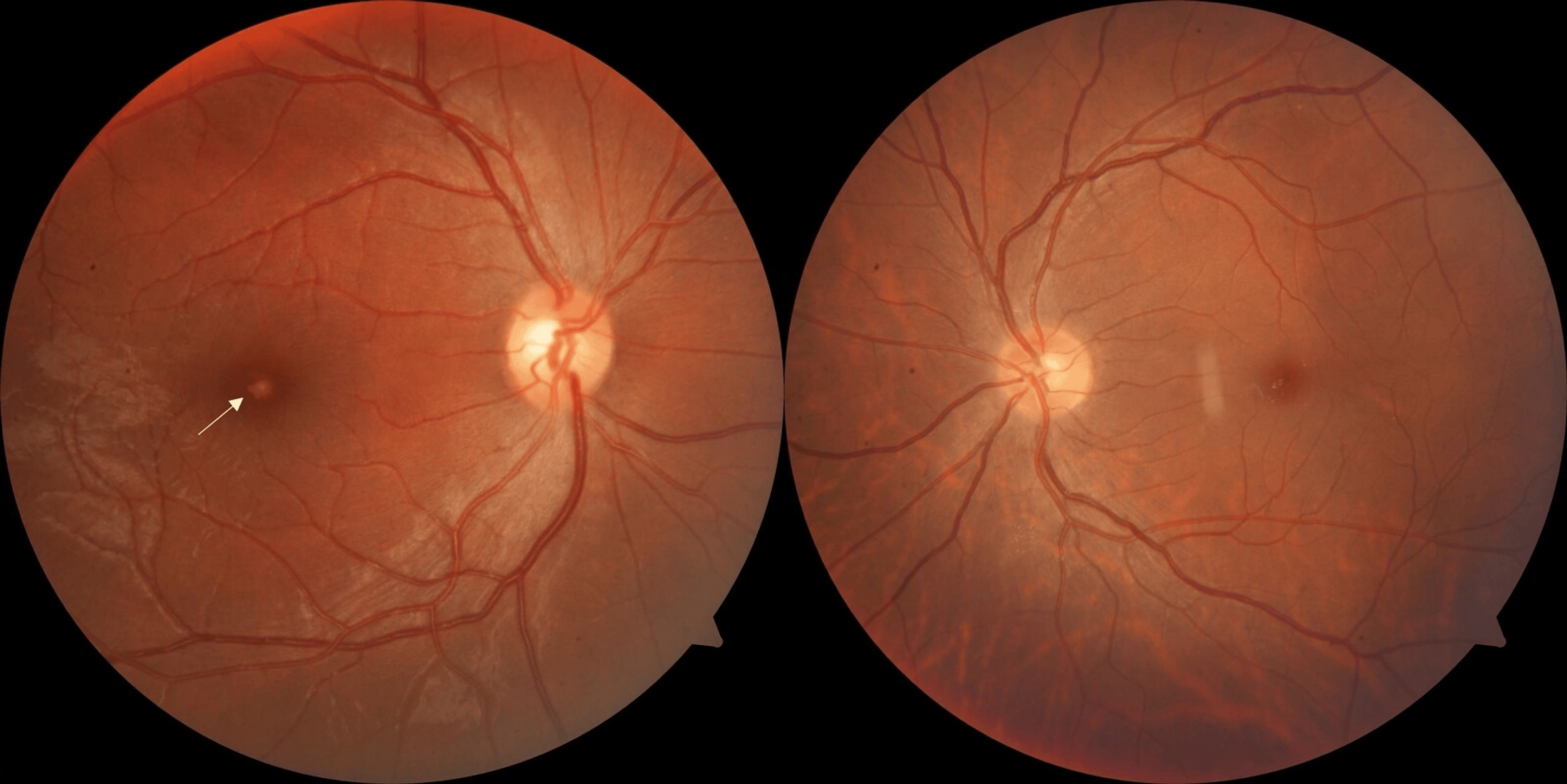
Fundus photographs showing a yellowish lesion in the fovea of the right eye (white arrow) and a normal left eye

Ocular examination revealed a visual acuity of 20/30 and N8 in the right eye and 20/20 and N6 (using standard Snellen's chart) in the left eye. The Amsler grid test revealed a central scotoma in the right eye, indicating a defect in central vision. The anterior segment of both eyes had no significant findings, with no signs of inflammation, cataract, or any other abnormalities. Fundus image of the right eye showed a well-defined yellowish lesion at the fovea. The left eye fundus was unremarkable (Figure 1).

**Figure 2:**
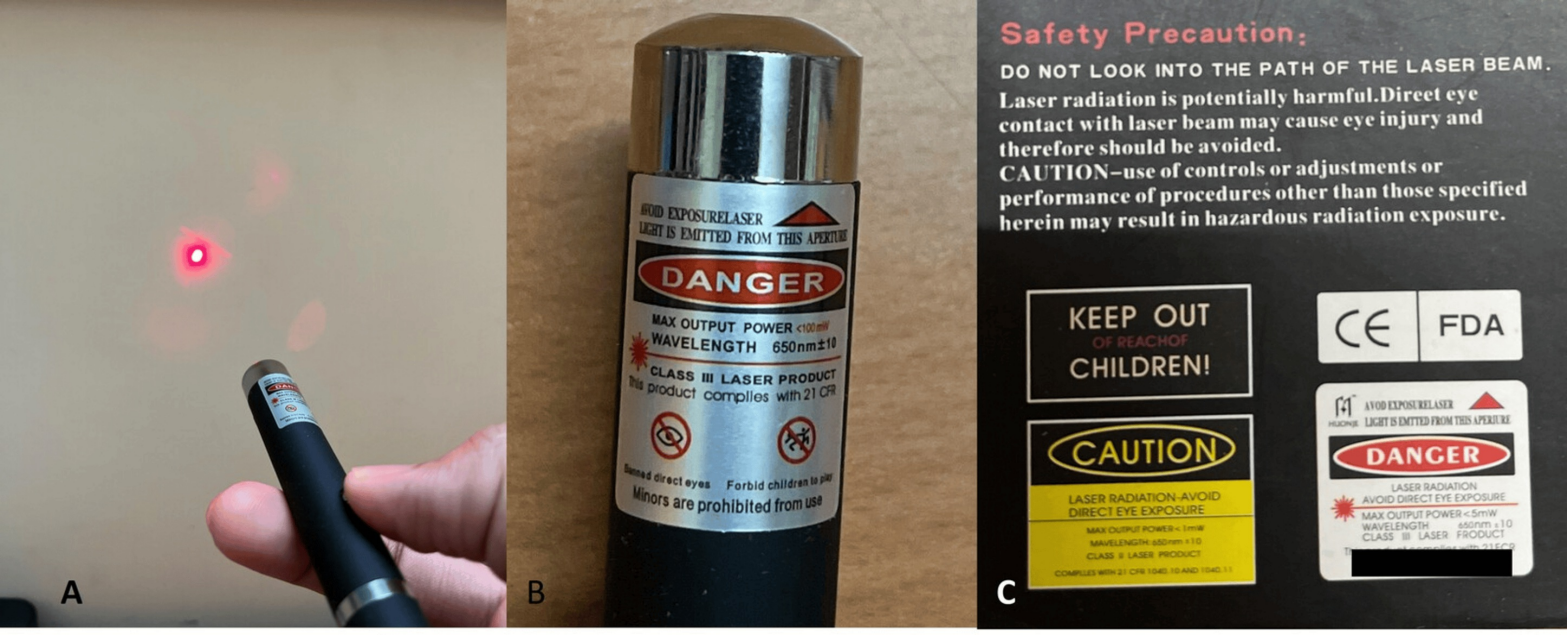
Image showing the red beam emitted by the pointer (A), the warning messages printed on the device (B), and the box (C)

We retrieved the laser pointer, and it emitted red light (wavelength 650 nm, power rating of 5 mW) (Figure 2).

**Figure 3:**
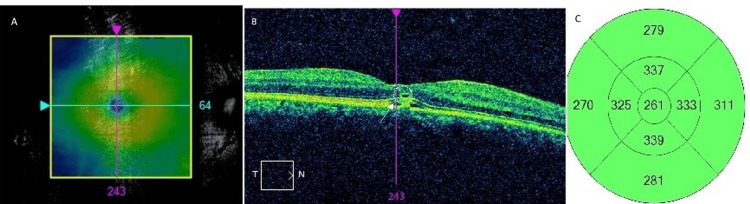
Optical coherence tomography of the right eye: (A) Location of the macular scan. (B) Features of photic maculopathy showing subfoveal hyper-reflectivity in the outer retinal layers, focal disruption of the inner segment-outer segment (IS-OS) junction, and a subfoveal hyporeflective cavity (white broken circle) and neurosensory detachment (white arrow). (C) Thickness map showing the normal thickness of the macula

Optical coherence tomography (OCT) was performed with Cirrus SD-OCT (Carl Zeiss Meditec®, Jena, Germany), and it showed subfoveal hyper-reflectivity in the outer retinal layers, focal disruption of the inner segment-outer segment (IS-OS) junction, and a subfoveal hyporeflective cavity, consistent with photic maculopathy. A shallow neurosensory detachment in the layers of the retina was seen (central macular thickness: 261 µm) (Figure 3).

**Figure 4:**
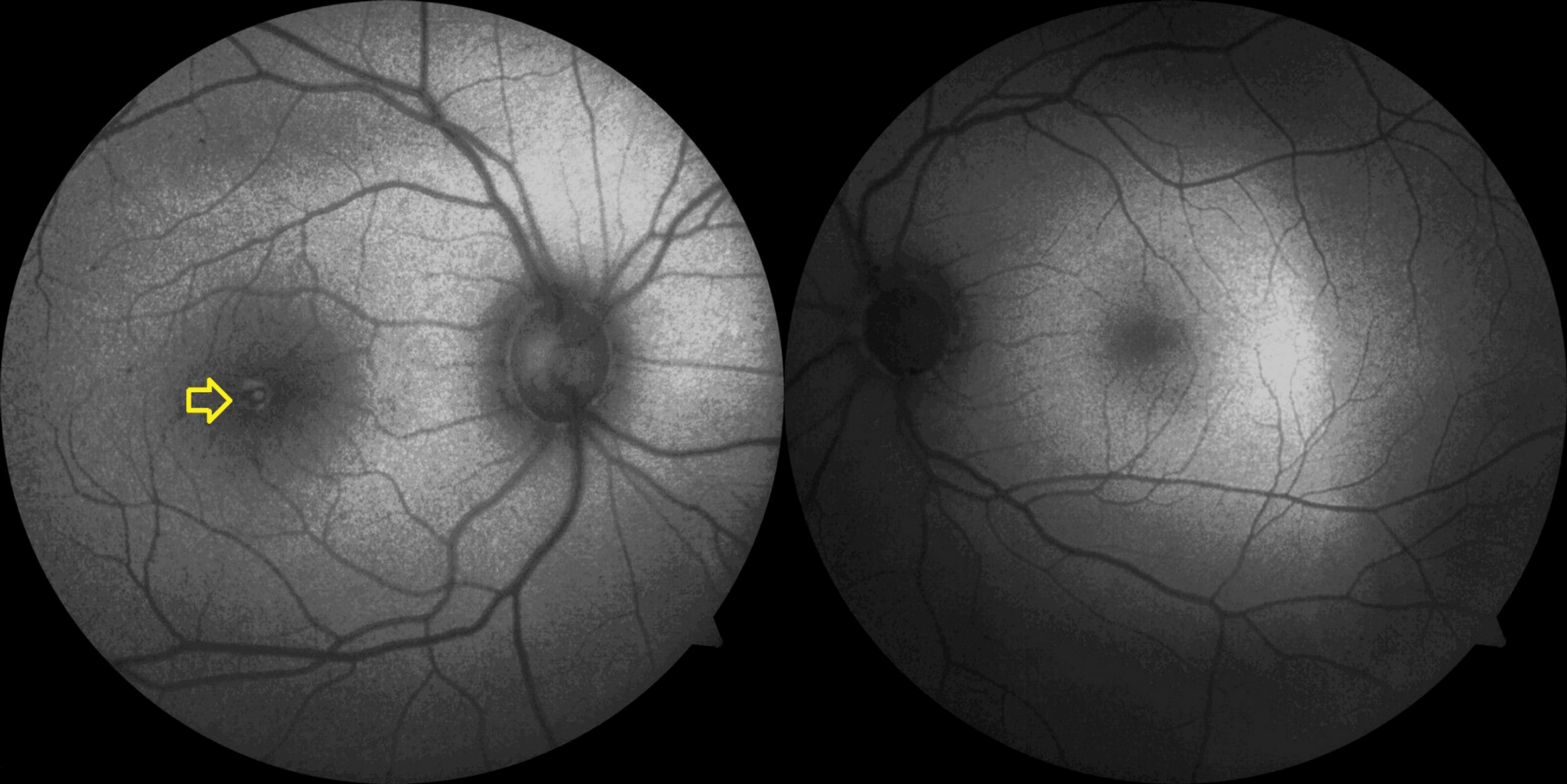
Short-wave autofluorescence showing a hypo-autofluorescent lesion at the fovea in the right eye (yellow arrow) and a normal left eye

Short-wave fundus autofluorescence (SW-FAF) or fundus autofluorescence detected a hyperfluorescent lesion, which was present at the fovea, corresponding to the area of injury. The center of the lesion appeared hypofluorescent, indicating RPE damage (Figure 4). 

Based on OCT findings, the differentials include (a) acute macular neuroretinopathy, (b) post-regressed central serous chorioretinopathy, (c) post-trauma Berlin's edema (regressed), (d) regressed punctate inner choroiditis, and (e) punctate outer retinal toxoplasmosis. Given the history of laser exposure and appearance of positive scotoma as perceived by the patient and temporal association, the diagnosis of photic maculopathy was made. 

**Figure 5:**
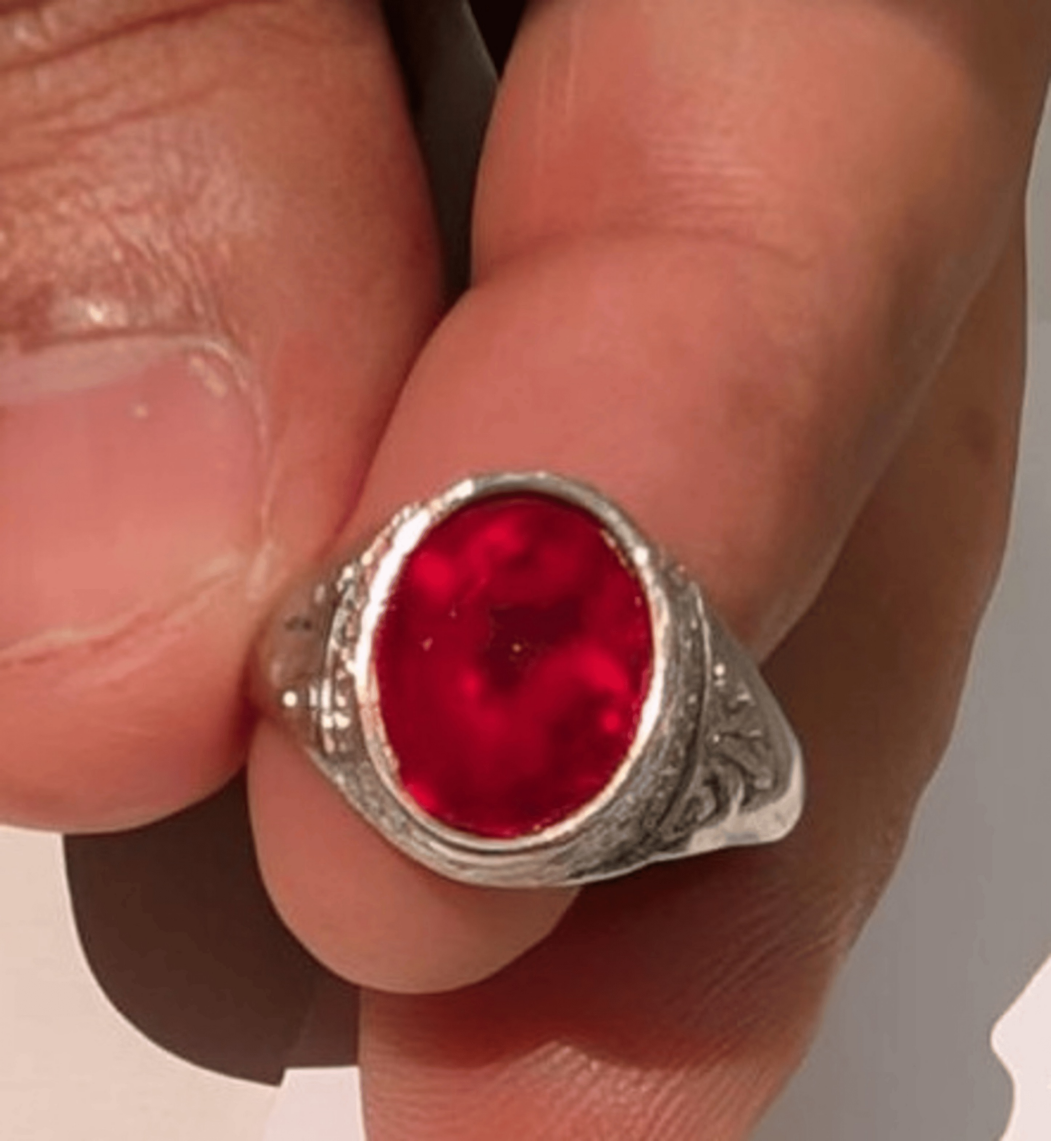
Pitting on stone tested by the patient with laser pointer

Presently, there is no approved treatment to reverse laser-induced retinal damage. However, management techniques aim to reduce inflammation, promote retinal healing, and prevent further complications. The patient was given a trial of oral prednisolone (1 mg/kg) for two weeks, as corticosteroids help reduce retinal inflammation and minimize secondary damage. At the end of six weeks, the patient's visual acuity had improved to 20/20 and N8 with a persistent scotoma. Fundus examination showed resolution of the yellowish lesion, replaced by subtle RPE alterations. Thereafter, he was lost to follow-up. Intrigued by the experience, our patient tried focusing the laser light on a red stone of his finger ring, and the laser caused pits on the stone (Figure 5).

## Discussion

The mechanism of action of the laser is based on three fundamental principles: absorption, spontaneous emission, and stimulated emission. The energy supplied excites the electron to a higher-energy state. This electron, after returning to the lower-energy state, emits a photon, which perpetuates the formation of a laser. The degree of injury depends upon the wavelength, duration of exposure, and composition of the retinal tissues. The location of the laser injury is important in determining the degree of disability. Laser light can cause a higher degree of visual loss in the case of foveal involvement [3]. The other determinants are the size of the pupil and the pigment in the RPE. 

Laser causes retinal damage by various mechanisms. Photothermal injury occurs when laser energy raises the temperature of retinal tissues [4], while photochemical injury results from free radical generation, particularly with shorter wavelengths like blue light [3]. Melanin, xanthophyll, and haemoglobin are key absorbers. Blue light, being of shorter wavelength, damages the inner retina, and red light, owing to its longer wavelength, tends to have deeper damage [5]. Thus, there is also a higher chance of developing choroidal neovascularization in eyes with damage from red lasers [6], warranting a longer follow-up. However, our patient was lost to follow-up after six weeks. Additionally, different pigments in the retina absorb light in various ways [3]. On exposure to laser light for prolonged periods, free radicals are generated. This causes damage to the retinal layers. This is mostly caused by blue light. RPE and photoreceptor injury are the usual consequences.

Photic or phototoxic maculopathy is a unilateral or bilateral condition that follows exposure to bright light, and it has multiple causes. Photic maculopathy has also been reported following the use of laser pointers emitting blue light [7], green light [8], and red light [9]. The diagnosis is usually straightforward, as there is a symptom of positive scotoma and visual disturbance following exposure to laser light and outer retinal damage that is best seen on OCT. The scotoma tends to persist, and treatment options for laser injury causing retinal damage are limited. Conservative management includes topical, periocular, and systemic steroids whose effectiveness is still not proven [10]. Nonsteroidal anti-inflammatory drugs (NSAIDs) can be used alternatively. Vascular endothelial growth factor (VEGF) inhibitors or surgery are suggested in severe cases when associated with choroidal neovascular membrane [11]. 

Blink reflex is a physiological protective reflex that involves the involuntary contraction of the orbicularis oculi muscle and protects the eye from external stimuli. It has a latency of 50-80 ms [12]. However, studies have shown that it is protective in only 15-20% of individuals following laser exposure [13].

Laser, hence, stands as a paradox in medicine. It can be a cause of injury by design, but when used with control and precision, it initiates a repair mechanism, transforming harm into restoration. Despite regulations, these laser pointers are easily available online, and due to a lack of awareness of the permanent visual disability caused by these devices, the incidence of laser-induced maculopathy is increasing. Considering the long-lasting effects of lasers on central vision, the easy availability of these pointers carries immense public health importance. Use of ultraviolet (UV)-protective eye wear should be encouraged in individuals using laser pointers and other devices that use laser. 

The device used in our case had warning labels and an information booklet. It was easily available for online purchase at a low cost. Despite labelling the devices with warning information, there have been reported incidences of laser pointer-induced ocular injuries. Highlighting and providing an image of the eye to educate the public about the ocular damages associated with laser might be helpful in further reiterating the facts about laser. The regulations controlling the sales of these devices should limit the power output to <1 mW for consumer use. There is a need to raise public awareness about this apparently harmless toy that can cause a permanent visual handicap. Further questionnaire-based studies can be performed among the consumers and dealers of this device to understand the level of knowledge about the harmful effects of lasers. The lack of long-term follow-up significantly limits our case. Also, as it is a description of a single case, no major conclusions can be drawn. 

## Conclusions

Photic maculopathy is a possibility in cases presenting with a sudden history of positive scotoma and exposure to laser light. Easy online availability and poor regulation of high-powered laser devices can contribute to a rise in the incidence of photic maculopathy. Management is expectant, with regular follow-up to monitor for complications of photic maculopathy.

## References

[1] González Martín-Moro J, Hernández Verdejo JL, Zarallo Gallardo J (2018). Photic maculopathy: a review of the literature (I). Arch Soc Esp Oftalmol (Engl Ed).

[2] Neffendorf JE, Hildebrand GD, Downes SM (2019). Handheld laser devices and laser-induced retinopathy (LIR) in children: an overview of the literature. Eye (Lond).

[3] Commiskey PW, Heisel CJ, Paulus YM (2019). Non-therapeutic laser retinal injury. Int J Ophthalmic Res.

[4] Barkana Y, Belkin M (2000). Laser eye injuries. Surv Ophthalmol.

[5] Zhang CX, Fan B, Chi J, Li YL, Jiao Q, Zhang ZY, Li GY (2024). Differences between long- and short-wavelength light-induced retinal damage and the role of PARP-1 in retinal injury induced by blue light. Exp Eye Res.

[6] Kaya M, Akbulut Yagci B (2021). Bilateral macular injury following red laser pointer exposure: a case report. Eur Eye Res.

[7] Rohring V, Rehmani A, Smith E, Smith E, Berg P (2019). Drone retinopathy. J Curr Ophthalmol.

[8] Mtanes K, Mimouni M, Zayit-Soudry S (2018). Laser pointer-induced maculopathy: more than meets the eye. J Pediatr Ophthalmol Strabismus.

[9] Zhang L, Zheng A, Nie H (2016). Laser-induced photic injury phenocopies macular dystrophy. Ophthalmic Genet.

[10] Timofte Zorila MM, Vitiello L, Lixi F, Coppola A, Cukurova F, Pellegrino A, Giannaccare G (2025). Photic retinopathy: diagnosis and management of this phototoxic maculopathy. Life (Basel).

[11] Amoroso F, Souied EH, Ansary MF, Astroz P, Mouallem-Bézière A, Pedinielli A, Miere A (2020). Optical coherence tomography angiography findings of choroidal neovascularization secondary to laser injury: a case report. Am J Ophthalmol Case Rep.

[12] Hackley SA, Johnson LN (2023). The photic blink reflex as an index of photophobia. Biol Psychol.

[13] Reidenbach HD, Dollinger K, Hofmann J (2002). Field trials with low power lasers concerning the blink reflex. Biomed Tech (Berl).

